# Supplementary material for: Promotion of neutralizing antibody-independent immunity to wild-type and SARS-CoV-2 variants of concern using an RBD-Nucleocapsid fusion protein
Source: Nat Commun. 2022 Aug 17;13:4831. doi: 10.1038/s41467-022-32547-y (PMC9382605; doi:10.1038/s41467-022-32547-y)
Supplement: Supplementary file 1 — Supplementary Information [file 41467_2022_32547_MOESM1_ESM.docx]

**Supplementary Figure 1**

**Supplementary Figure 1. Human antibody and IFN-γ responses to N and RBD polypetides. a,** SDS-PAGE of purified SpiN, RBD, N and S proteins. **b, c,** Western blots of purified SpiN, RBD and N proteins using rabbit polyclonal anti-N and a mouse monoclonal anti-RBD antibodies are shown in panels. Data are representative of two independent experiments. **d-f,** The levels of IgG antibodies specific for S, N and RBD proteins found in sera from healthy controls (HC), vaccinated (Vacc) and convalescents (Conv), as indicated. Data are presented as mean +/- SEM. **g-i,** The IFN-γ response, respectively, to the S, N and RBD proteins by the PBMCs from HC, vaccinated and convalescent individuals. The lines link the levels of IFN-γ produced by PBMCs cultured in the absence and presence of antigens, as indicated. The number of individuals used in these experiments was 9 controls, 33 vaccinated and 13 convalescents. **j,** Cytokine measurements in the supernatants of PBMCs stimulated with SpiN, data are presented as mean +/- SEM. IgG antibodies measurements **(d-f)** were analyzed through Two-way ANOVA followed by Dunn’s multiple comparisons test. Statistical analyses of IFN-γ production and flow cytometry were performed using Wilcoxon-matched pairs signed rank. “ns” indicates that difference is not statistically significant. Cytokine measurements were analyzed by Two-way ANOVA followed by Tukey’s multiple comparisons test. * P < 0.05, ** P < 0.01 and *** P < 0.001 and **** P < 0.0001.

**Supplementary Figure 2**

**
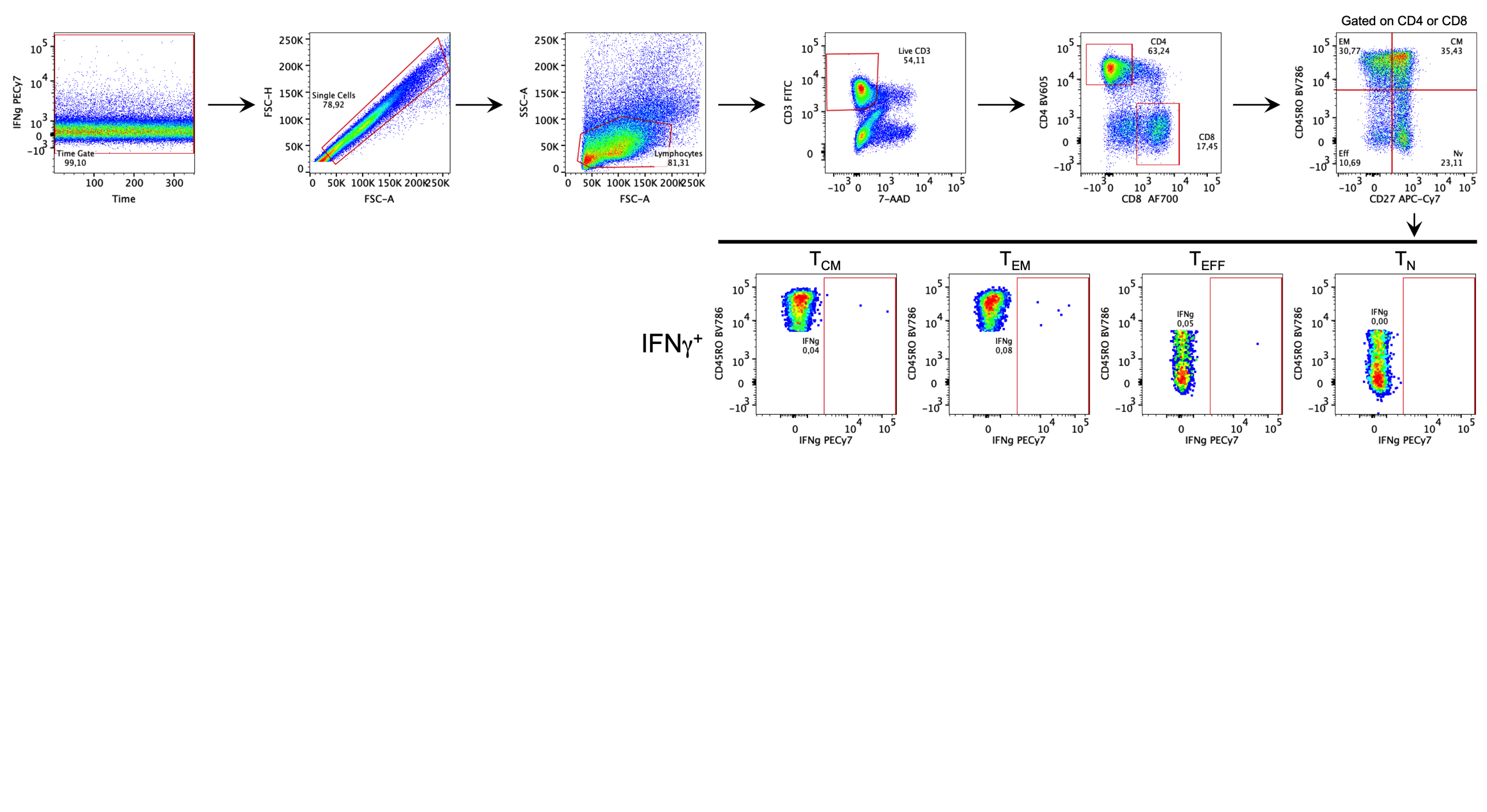
**

**Supplementary Figure 2. Workflow and gating strategy for flow cytometry of human PBMCs.** Upper panel shows the gating strategy to identify CD4+ and CD8+ T cells, and memory subpopulations defined by the expression of CD45RO and CD27. Lower panels with representative plots showing detection of antigen-induced activation measured by intracellular staining of IFN-γ after SpiN stimulation. Upregulation of cytokines were analyzed separately among CD4^+^ or CD8^+^ T cells.

**Supplementary Figure 3**


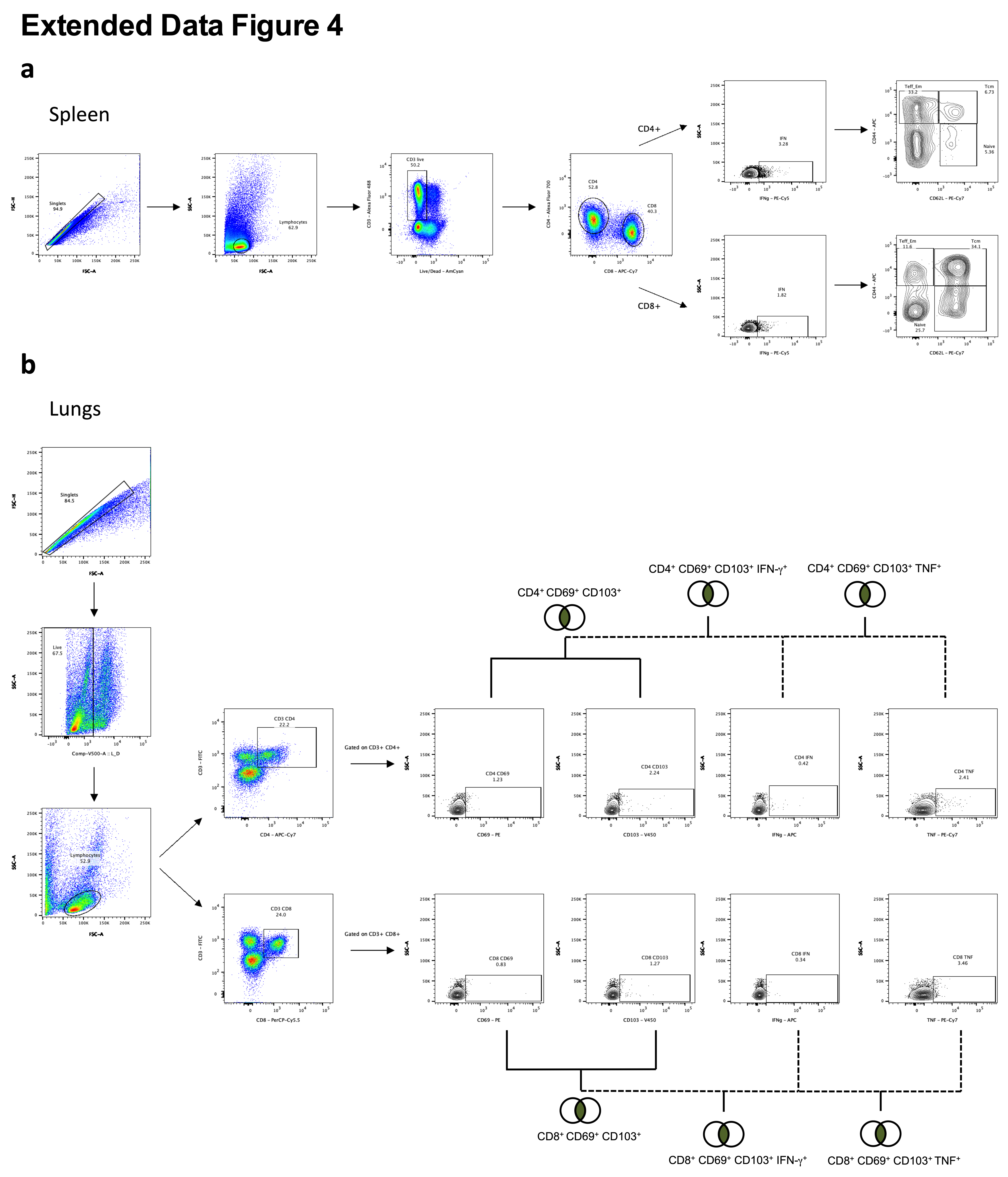


**Supplementary Figure 3. Gating strategy of flow cytometry performed in spleens and lungs.** **a,b,** Thirty days post vaccination, SpiN-immunized mice had their spleen **(a)** or lungs **(b)** harvested. The cells were isolated, stimulated with SpiN and then stained for memory T cell characterization. **a,** Splenocytes were gated for singlets (FSC-H X FSC-A), lymphocytes (SSC-A x FSC-A), live CD3+ (CD3 x Live/Dead), CD4+ or CD8+ (SSC-A x CD4, SSC-A x CD8), IFN-γ+ (SSC-A x IFN-γ) and with CD44 x CD62L they were separated into naïve (CD44- CD62L+), effector/effector memory (CD44+ CD62L-) and central memory (CD44+ CD62L+). **b,** Lungs cells were gated for singlets (FSC-H X FSC-A), live (SSC-A x Live/Dead), lymphocytes (SSC-A x FSC-A), CD3+ CD4+ or CD3+ CD8+ (CD3 x CD4, CD3 x CD8). CD4+ or CD8+ T lymphocytes were characterized in CD69+ CD103+ tissue-resident memory (Trm) cells by Boolean analysis “AND gate” (CD69+ and CD103+), Trm IFN-γ+ (CD69+ CD103+ and IFN-γ+), Trm TNF+ (CD69+ CD103+ and TNF+).

**Supplementary Figure 4**

**
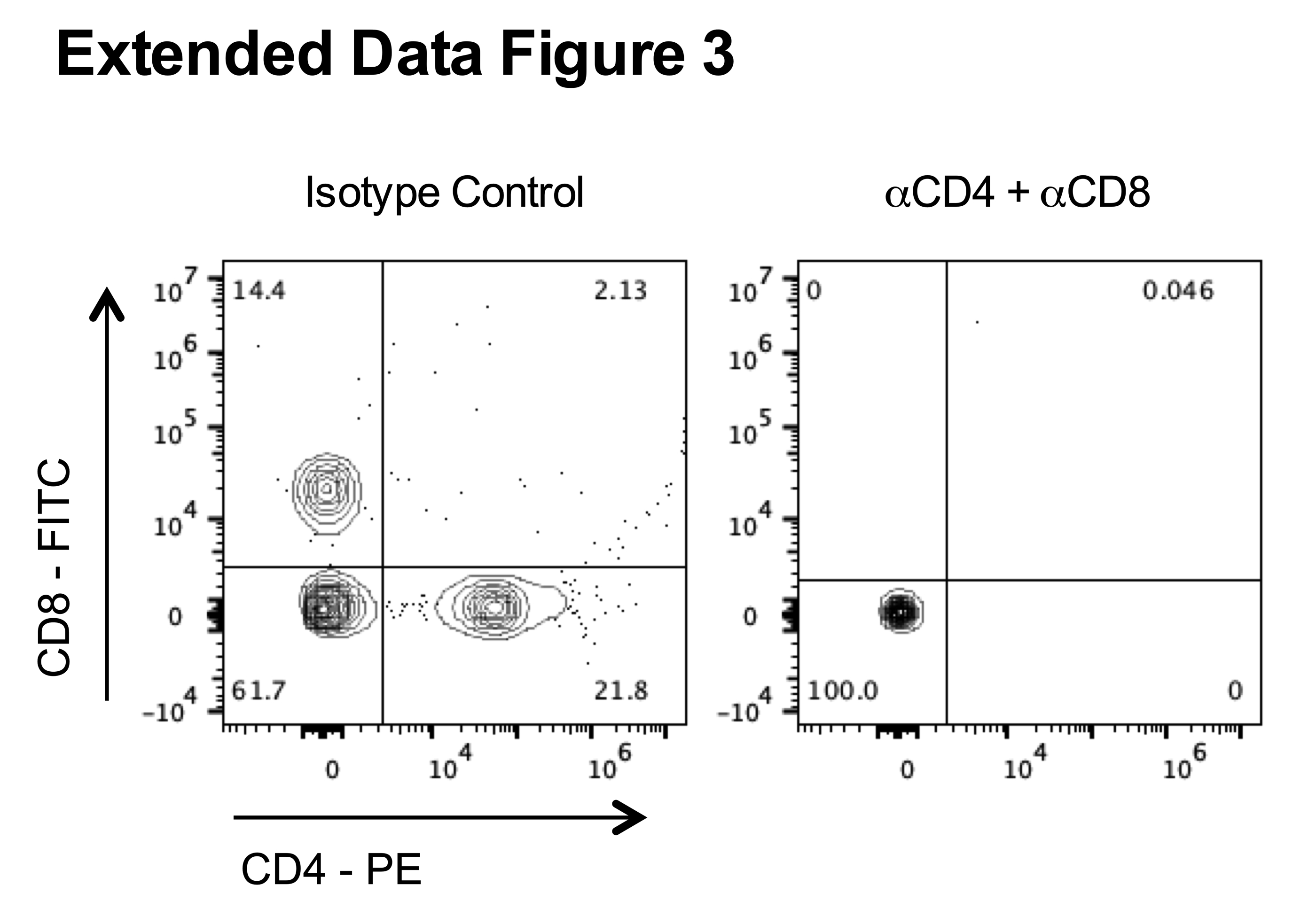
**

**Supplementary Figure 4. CD4+ and CD8+ T cell depletion with GK1.5 and 2.43 monoclonal antibodies.** Animals were treated on days -3, -2 and -1 with isotype control (anti-KLH), anti-CD4, anti-CD8 or anti-CD4 and anti-CD8. Depletion was confirmed by flow cytometry of total blood cells, in which cells were gated for singlets (FSC-H x FSC-A), lymphocytes (SSC-A x FSC-A) and then CD8-FITC x CD4-PE.

**Supplementary Table 1 -** Amino acid sequences of putative epitopes from RBD and N proteins found by *in silico* epitope prediction and their respective binding scores for HLA-ABC.

|  | **Peptide** | **HLA-ABC** | **Percentile Rank (IEDB)** |
| --- | --- | --- | --- |
| **RBD** | KLNDLCFTNV | A*A*02:01 | 0.24 |
|  | FELLHAPATV | A*A*02:01; B*40:01 | 0.26; 0.95 |
|  | KLPDDFTGCV | A*A*02:01 | 0.23 |
|  | LYNSASFSTF | A*24:02 | 0.03 |
|  | YNSASFSTFK | A*11:01 | 0.33 |
|  | NLDSKVGGNY | A*01:01 | 0.1 |
|  | RQIAPGQTGK | A*03:01; A*11:01 | 0.03; 0.13 |
|  | QPYRVVVLSF | B*0702 | 0.29 |
|  | FERDISTEIY | B*44:02 | 0.24 |
|  | TESNKKFLPF | B*44:02 | 0.25 |
| **Nucleocapsid** | YLGTGPEAGL | A*02:01 | 0.7 |
|  | WLTYTGAIKL | A*02:01 | 0.99 |
|  | LLLLDRLNQL | A*02:01; B*08:01 | 0.33; 0.99 |
|  | ADLDDFSKQL | A*02:01 | 0.33; |
|  | VLQLPQGTTL | A*02:01 | 0.57 |
|  | QFAPSASAFF | A*24:02 | 0.16 |
|  | AQFAPSASAF | A*24:02; B*40:01; B*44:02; | 0.46; 1.0; 0.83 |
|  | YKHWPQIAQF | A*24:02 | 0.65 |
|  | SSPDDQIGYY | A*01:01 | 0.01 |
|  | VTPSGTWLTY | A*01:01 | 0.09 |
|  | GTGPEAGLPY | A*01:01 | 0.21 |
|  | NSSPDDQIGY | A*01:01 | 0.11 |
|  | QGTTLPKGFY | A*01:01 | 0.56 |
|  | DLSPRWYFYY | A*01:01 | 0.79 |
|  | KTFPPTEPKK | A*03:01; A*11:01 | 0.01; 0.01 |
|  | YKTFPPTEPK | A*03:01; A*11:01 | 0.14; 0.05 |
|  | ATEGALNTPK | A*03:01; A*11:01 | 0.7; 0.17 |
|  | KLDDKDPNFK | A*03:01; A*11:01 | 0.21; 0.55 |
|  | LLNKHIDAYK | A*03:01 | 0.4 |
|  | RIRGGDGKMK | A*03:01 | 0.38 |
|  | KFPRGQGVPI | B*07:02 | 0.04 |
|  | NPANNAAIVL | B*07:02 | 0.53 |
|  | APSASAFFGM | B*07:02 | 0.62 |
|  | RPQGLPNNTA | B*07:02 | 0.25 |
|  | RNPANNAAIV | B*07:02 | 0.86 |
|  | FPRGQGVPIN | B*07:02 | 0.77 |
|  | RGPEQTQGNF | B*07:02 | 0.6 |
|  | SASAFFGMSR | A*11:01 | 0.74 |
|  | KKSAAEASKK | A*11:01 | 0.75 |
|  | DPNFKDQVIL | B*08:01 | 0.75 |
|  | MEVTPSGTWL | B*40:01; B*44:02 | 0.17; 0.55 |
|  | GMEVTPSGTW | B*44:02 | 0.05 |

**Supplementary Table 2 -** Amino acid sequences of putative epitopes from RBD and N proteins found by *in silico* epitope prediction and their respective binding scores for HLA-DR.

|  | **Peptide Core** | **HLA-DR** | **Percentile Rank (NetMHCII)** |
| --- | --- | --- | --- |
| **RBD** | FELLHAPAT | B1*01:01 | 0.82 |
|  | FNGLTGTGV | B1*01:01 | 0.26 |
|  | IRGDEVRQI | B1*03:01 | 0.73 |
|  | FERDISTEI | B1*03:05 | 0.24 |
|  | YGFQPTNGV | B1*04:01 | 0.59 |
|  | FQPTNGVGY | B1*07:01; B1*09:01 | 0.34; 0.87 |
|  | VIAWNSNNL | B1*15:01; B1*15:03; B1*13:01 | 0.06; 0.12; 0.34 |
|  | IVRFPNITN | B1*15:01; B1*15:03 | 0.52; 0.62 |
| **Nucleocapsid** | VTLLPAADL | B1*01:01; B1*12:01 | 0.47; 0.23 |
|  | FYAEGSRGG | B1*01:01; B1*01:03; B1*04:01 | 0.63; 027; 0.15 |
|  | WFTALTQHG | B1*04:01 | 0.11 |
|  | IKLDDKDPN | B1*03:01; B1*03:05 | 0.11; 021 |
|  | LTYTGAIKL | B1*07:01; B1*09:01 | 0.02; 011 |
|  | YNVTQAFGR | B1*07:01 | 0.85 |
|  | FAPSASAFF | B1*07:01 | 0.86 |
|  | VILLNKHID | B1*08:01; B1*08:03; B1*11:01; B1*12:01; B1*14:01 | 0.02; 0.04; 0.39; 0.73; 0.27 |
|  | YTGAIKLDD | B1*08:01; B1*12:01 | 0.16; 0.12 |
|  | IGYYRRATR | B1*13:01 | 0.02 |
|  | IDAYKTFPP | B1*15:01 | 0.03 |

**Supplementary Table 3 -** Amino acid sequences of putative epitopes from RBD and N proteins found by *in silico* epitope prediction and their respective binding scores for mouse MHC-I.

|  | **Peptide** | **MHC-I** | **Percentile Rank (IEDB)** |
| --- | --- | --- | --- |
| **RBD** | IVRFPNITNL | H-2K^b^; H-2D^b^ | 0.33; 0.38 |
|  | VGYQPYRVVV | H-2K^b^ | 0.98 |
|  | YSVLYNSASF | H-2D^b^ | 0.17 |
|  | STPCNGVEGF | H-2D^b^ | 0.3 |
|  | SKVGGNYNYL | H-2D^b^ | 0.37 |
| **Nucleo capsid** | LSPRWYFYYL | H-2K^b^ | 0.82 |
|  | ALALLLLDRL | H-2D^b^ | 1.1 |
|  | TRNPANNAAI | H-2D^b^ | 1.2 |
|  | VLQLPQGTTL | H-2D^b^ | 1.2 |

**Supplementary Table 4 -** Amino acid sequences of putative epitopes from RBD and N proteins found by *in silico* epitope prediction and their respective binding scores for mouse MHC-II.

|  | **Peptide** | **MHC-II** | **Percentile Rank (NetMHCII)** |
| --- | --- | --- | --- |
| **RBD** | LSFELLHAPATVCGP | H-2IA^b^ | 1.7 |
|  | VLSFELLHAPATVCG | H-2IA^b^ | 1.8 |
| **Nucleocapsid** | WPQIAQFAPSASAFF | H-2IA^b^ | 0.12 |
|  | PQIAQFAPSASAFFG | H-2IA^b^ | 0.15 |
|  | QIAQFAPSASAFFGM | H-2IA^b^ | 0.17 |
|  | HWPQIAQFAPSASAF | H-2IA^b^ | 0.3 |
|  | IAQFAPSASAFFGMS | H-2IA^b^ | 0.4 |
|  | KHWPQIAQFAPSASA | H-2IA^b^ | 0.6 |
|  | AQFAPSASAFFGMSR | H-2IA^b^ | 1.2 |
|  | RWYFYYLGTGPEAGL | H-2IA^b^ | 1.5 |
|  | PRWYFYYLGTGPEAG | H-2IA^b^ | 1.5 |
|  | WYFYYLGTGPEAGLP | H-2IA^b^ | 1.8 |
|  | YFYYLGTGPEAGLPY | H-2IA^b^ | 1.9 |

**Supplementary Table 5** - Healthy donors, COVID-19 convalescent and CoronaVac vaccinated individuals enrolled in our study.

| **Cod** | **Group** | **Sex** | **Age** | **Days between vaccination and sampling** | **Sampling date** | **Vaccination day (2^nd^ dose)** | **Date of COVID PCR^+^** | **Vaccine** |
| --- | --- | --- | --- | --- | --- | --- | --- | --- |
| **804-R** | Conv | Female | 22 | NA | 03/21 | NA | 09/20 | NA |
| **805-R** | Conv | Female | 29 | NA | 03/21 | NA | 03/21 | NA |
| **806-R** | Conv | Female | 20 | NA | 03/21 | NA | 01/21 | NA |
| **807-R** | Conv | Female | 33 | NA | 03/21 | NA | 01/21 | NA |
| **808-R** | Conv | Male | 23 | NA | 04/21 | NA | 03/21 | NA |
| **809-R** | Conv | Female | 44 | NA | 04/21 | NA | 12/20 | NA |
| **810-R** | Conv | Female | 39 | NA | 04/21 | NA | 11/20 | NA |
| **811-R** | Conv | Female | 32 | NA | 04/21 | NA | 03/21 | NA |
| **812-R** | Conv | Female | 38 | NA | 04/21 | NA | 12/20 | NA |
| **813-R** | Conv | Male | 26 | NA | 04/21 | NA | 09/20 | NA |
| **814-R** | Conv | Female | 59 | NA | 04/21 | NA | 03/21 | NA |
| **817-R** | Conv | Female | 25 | NA | 05/21 | NA | 04/21 | NA |
| **820-R** | Conv | Female | 35 | NA | 05/21 | NA | 04/21 | NA |
| **038-0** | HD | Female | 40 | NA | 06/21 | NA | 06/20 | NA |
| **063-0** | HD | Female | 60 | NA | 05/21 | NA | NA | NA |
| **104-0** | HD | Female | 18 | NA | 06/21 | NA | NA | NA |
| **105-0** | HD | Male | 25 | NA | 06/21 | NA | NA | NA |
| **904-H** | HD | Female | 20 | NA | 03/21 | NA | NA | NA |
| **906-H** | HD | Male | 28 | NA | 03/21 | NA | NA | NA |
| **908-H** | HD | Male | 48 | NA | 03/21 | NA | NA | NA |
| **909-H** | HD | Male | 38 | NA | 03/21 | NA | NA | NA |
| **913-H** | HD | Female | 38 | NA | 04/21 | NA | NA | NA |
| **001-A** | Vacc | Female | 25 | 33 | 03/21 | 02/21 | NA | Coronavac |
| **002-A** | Vacc | Female | 27 | 33 | 03/21 | 02/21 | NA | Coronavac |
| **003-A** | Vacc | Female | 26 | 30 | 03/21 | 02/21 | NA | Coronavac |
| **004-A** | Vacc | Male | 56 | 32 | 03/21 | 02/21 | NA | Coronavac |
| **005-A** | Vacc | Male | 31 | 27 | 03/21 | 02/21 | NA | Coronavac |
| **006-A** | Vacc | Female | 39 | 29 | 03/21 | 02/21 | NA | Coronavac |
| **007-A** | Vacc | Female | 28 | 29 | 03/21 | 02/21 | NA | Coronavac |
| **008-A** | Vacc | Female | 27 | 29 | 03/21 | 02/21 | NA | Coronavac |
| **009-A** | Vacc | Female | 30 | 28 | 03/21 | 02/21 | NA | Coronavac |
| **010-A** | Vacc | Female | 36 | 31 | 03/21 | 02/21 | 08/20 | Coronavac |
| **017-A** | Vacc | Female | 27 | 41 | 03/21 | 02/21 | NA | Coronavac |
| **018-A** | Vacc | Female | 26 | 45 | 03/21 | 02/21 | NA | Coronavac |
| **019-A** | Vacc | Female | 27 | 38 | 03/21 | 02/21 | NA | Coronavac |
| **020-A** | Vacc | Female | 37 | 38 | 03/21 | 02/21 | NA | Coronavac |
| **021-A** | Vacc | Female | 39 | 48 | 0321 | 02/21 | NA | Coronavac |
| **022-A** | Vacc | Female | 70 | 34 | 03/21 | 02/21 | 03/20 | Coronavac |
| **023-A** | Vacc | Female | 31 | 36 | 03/21 | 02/21 | NA | Coronavac |
| **024-A** | Vacc | Female | 38 | 47 | 03/21 | 02/21 | NA | Coronavac |
| **025-A** | Vacc | Female | 39 | 46 | 03/21 | 02/21 | NA | Coronavac |
| **026-A** | Vacc | Female | 48 | 43 | 03/21 | 02/21 | NA | Coronavac |
| **027-A** | Vacc | Female | 50 | 48 | 03/21 | 02/21 | NA | Coronavac |
| **028-A** | Vacc | Female | 54 | 42 | 03/21 | 02/21 | NA | Coronavac |
| **029-A** | Vacc | Female | 42 | 42 | 03/21 | 02/21 | NA | Coronavac |
| **030-A** | Vacc | Female | 28 | 43 | 03/21 | 02/21 | NA | Coronavac |
| **031-A** | Vacc | Male | 60 | 44 | 04/21 | 02/21 | NA | Coronavac |
| **034-A** | Vacc | Male | 47 | 45 | 04/21 | 02/21 | NA | Coronavac |
| **035-A** | Vacc | Male | 39 | 45 | 04/21 | 02/21 | 11/20 | Coronavac |
| **036-A** | Vacc | Female | 44 | 43 | 04/21 | 02/21 | NA | Coronavac |
| **037-A** | Vacc | Female | 28 | 47 | 04/21 | 02/21 | 11/20 | Coronavac |
| **039-A** | Vacc | Female | 29 | 53 | 04/21 | 02/21 | 11/20 | Coronavac |
| **040-A** | Vacc | Female | 27 | 54 | 04/21 | 02/21 | NA | Coronavac |
| **041-A** | Vacc | Male | 60 | 54 | 04/21 | 02/21 | NA | Coronavac |
| **042-A** | Vacc | Male | 31 | 49 | 04/21 | 02/21 | NA | Coronavac |

Abbreviations: NA, not applicable; Conv, convalescent; HD, healthy donors; Vacc, vaccinated.

**Supplementary Table 6** - Primer sequences used to quantify cytokine and chemokine mRNAs by RT-PCR.

| **Gene** | **Primer Sequences** | |
| --- | --- | --- |
| **IL-1β** | F 5’ ACCTGTCCTGTGTAATGAAAGACG 3’ | R 5’ TGGGTATTGCTTGGGATCCA 3’ |
| **IFN-α** | F 5’ GTTCAAGTCTCTGTCCCCAAAA 3’ | R 5’ GTGGGAACTGCACCTGATGT 3’ |
| **IFN-β** | F 5’ CAGCTCCAAGAAAGGACGAAC 3’ | R 5’ GGCAGTGTAACTCTTCTGCAT 3’ |
| **TNF** | F 5’ CCCTCACACTCAGATCATCTTCT 3’ | R 5’ GCTACGACGTGGGCTACAG 3’ |
| **IL-6** | F 5’ TGTTCTCTGGGAAATCGTGGA 3’ | R 5’ AAGTGCATCATCGTTGTTCATACA 3’ |
| **IL-4** | F 5’ TCATCGGCATTTTGAACGAG 3’ | R 5’ CGTTTGGCACATCCATCTCC 3’ |
| **IL-5** | F 5’ AAAGAGAAGTGTGGCGAGGAGA 3’ | R 5’ CACCAAGGAACTCTTGCAGGTAA 3’ |
| **CCL2** | F 5’ TGGCTCAGCCAGATGCAGT 3’ | R 5’ TTGGGATCATCTTGCTGGTG 3’ |
| **CCL5** | F 5’ CAAGTGCTCCAATCTTGCAGTC 3’ | R 5’ TTCTCTGGGTTGGCACACAC 3’ |
| **CCL17** | F 5’ CAGGGATGCCATCGTGTTTC 3’ | R 5’ CACCAATCTGATGGCCTTCTT 3’ |
| **CXCL9** | F 5’ AATGCACGATGCTCCTGCA 3’ | R 5’ GGTCTTTGAGGGATTTGTAGTG 3’ |
| **CXCL10** | F 5’ GCCGTCATTTTCTGCCTCA 3’ | R 5’ CGTCCTTGCGAGAGGGATC 3' |
