# Supplementary material for: Promotion of neutralizing antibody-independent immunity to wild-type and SARS-CoV-2 variants of concern using an RBD-Nucleocapsid fusion protein
Source: Nat Commun. 2022 Aug 17;13:4831. doi: 10.1038/s41467-022-32547-y (PMC9382605; doi:10.1038/s41467-022-32547-y)
Supplement: Supplementary file 3 — Reporting Summary [file 41467_2022_32547_MOESM3_ESM.pdf]

nature research

Corresponding author(s): Ricardo T. Gazzinelli

Last updated by author(s): Jul 4, 2022

Reporting Summary

Nature Research wishes to improve the reproducibility of the work that we publish. This form provides structure for consistency and transparency in reporting. For further information on Nature Research policies, see our Editorial Policies and the Editorial Policy Checklist.

Statistics

For all statistical analyses, confirm that the following items are present in the figure legend, table legend, main text, or Methods section.

n/a	Confirmed	
☐	☒	The exact sample size (*n*) for each experimental group/condition, given as a discrete number and unit of measurement
☐	☒	A statement on whether measurements were taken from distinct samples or whether the same sample was measured repeatedly
☐	☒	The statistical test(s) used AND whether they are one- or two-sided<br>*Only common tests should be described solely by name; describe more complex techniques in the Methods section.*
☐	☒	A description of all covariates tested
☐	☒	A description of any assumptions or corrections, such as tests of normality and adjustment for multiple comparisons
☐	☒	A full description of the statistical parameters including central tendency (e.g. means) or other basic estimates (e.g. regression coefficient) AND variation (e.g. standard deviation) or associated estimates of uncertainty (e.g. confidence intervals)
☐	☒	For null hypothesis testing, the test statistic (e.g. *F*, *t*, *r*) with confidence intervals, effect sizes, degrees of freedom and *P* value noted<br>*Give P values as exact values whenever suitable.*
☒	☐	For Bayesian analysis, information on the choice of priors and Markov chain Monte Carlo settings
☒	☐	For hierarchical and complex designs, identification of the appropriate level for tests and full reporting of outcomes
☒	☐	Estimates of effect sizes (e.g. Cohen's *d*, Pearson's *r*), indicating how they were calculated

Our web collection on statistics for biologists contains articles on many of the points above.

Software and code

Policy information about availability of computer code

Data collection	Western Blot Images: ImageLab™ Touch Software V6.0.1 (Bio-Rad)<br>Flow Cytometry: BD FACSDIVA V8.0.1<br>ELISA:SOFTmaxPRO V4.3.1 LS<br>IFA: ZEN imaging software, ZEISS V1.1.2.0
Data analysis	Needle plot:<br>- MUSCLE tool<br>- R packages: mustneedle and ggtree<br>- IQ-TREE<br><br>Western Blot Images: ImageStudio™ V5.2<br>Flow Cytometry: FlowJo V10.5.3<br>Statistics: GraphPad Prism V6.0<br>IFA: ImageJ V2.1

For manuscripts utilizing custom algorithms or software that are central to the research but not yet described in published literature, software must be made available to editors and reviewers. We strongly encourage code deposition in a community repository (e.g. GitHub). See the Nature Research guidelines for submitting code & software for further information.

Data

Policy information about availability of data

All manuscripts must include a data availability statement. This statement should provide the following information, where applicable:

- Accession codes, unique identifiers, or web links for publicly available datasets
- A list of figures that have associated raw data
- A description of any restrictions on data availability

The authors declare that all data supporting the findings of this study are available within the paper and its supplementary information files. For epitope prediction of Spike (6VSB DOI 10.2210/pdb6VSB/pdb) and Nucleocapsid (7SD4_1 10.2210/pdb7SD4/pdb) proteins, we used The Immune Epitope Database (IEDB). For the needle plot, we used Spike and Nucleocapsid amino acid sequences recovered from the alignment of the reference genomes of Alpha (MZ344997 https://www.ncbi.nlm.nih.gov/nuccore/MZ344997.1/), Beta (MW598419.1 https://www.ncbi.nlm.nih.gov/nuccore/MW598419.1/), Delta (MZ359831.1 https://www.ncbi.nlm.nih.gov/nuccore/MZ359831.1), Gamma (MZ169911.1 https://www.ncbi.nlm.nih.gov/nuccore/MZ169911.1/), Omicron BA.1 (OL672836.1 https://www.ncbi.nlm.nih.gov/nuccore/OL672836.1/), Omicron BA.2 (PRJNA784038 https://www.ncbi.nlm.nih.gov/bioproject/PRJNA784038) and the original lineage (B1 – Wuhan – EPI_ISL_402123 https://www.epicov.org/epi3/frontend#4c5e54). Source data for figs 1f,g; 2b-p; 3a-f; 4a-g; 5a-c; 6a-e,g-o; 7a-h; 8a-d are provided with the paper. If any more information is needed, data are available from the corresponding author upon reasonable request.

Field-specific reporting

Please select the one below that is the best fit for your research. If you are not sure, read the appropriate sections before making your selection.

☒ Life sciences ☐ Behavioural & social sciences ☐ Ecological, evolutionary & environmental sciences

For a reference copy of the document with all sections, see nature.com/documents/nr-reporting-summary-flat.pdf

Life sciences study design

All studies must disclose on these points even when the disclosure is negative.

Sample size	The group sizes for experiments with mice, hamsters and human samples were determined by power calculations statistical analysis.
Data exclusions	No data were excluded from the analysis.
Replication	The experiments were repeated at least twice, if the statistical significance was P<0.05. All attempts at replication were successful.
Randomization	In mice, hamsters and human experiments we used age and sex-matched individual in the control groups.
Blinding	Except for the histopathology description that was performed by two independent observers, we didn't find the need for blinding analysis in the other experiments because data was objective and phenotypes were very distinct.

Reporting for specific materials, systems and methods

We require information from authors about some types of materials, experimental systems and methods used in many studies. Here, indicate whether each material, system or method listed is relevant to your study. If you are not sure if a list item applies to your research, read the appropriate section before selecting a response.

Materials & experimental systems

n/a	Involved in the study	
☐	☒	Antibodies
☐	☒	Eukaryotic cell lines
☒	☐	Palaeontology and archaeology
☐	☒	Animals and other organisms
☐	☒	Human research participants
☒	☐	Clinical data
☒	☐	Dual use research of concern

Methods

n/a	Involved in the study	
☒	☐	ChIP-seq
☐	☒	Flow cytometry
☒	☐	MRI-based neuroimaging

Antibodies

Antibodies used

- Anti-Mouse CD3 PerCP-Cy5.5 (BD, clone 145-2C11, Cat 551163, Lot 2209995)
- Anti-Mouse CD4 Alexa Fluor 700 (Invitrogen, clone KG1.5, Cat 56-0041-82, Lot 2012530)
- Anti-Mouse CD8 APC/Cy7 (Biolegend, clone 53-6.7, Cat 100714, Lot B217172)
- Anti-Mouse CD44 APC (Invitrogen, clone IM7, Cat 17-0441-82, Lot 2023951)
- Anti-Mouse IFNg APC (BD, clone XMG1.2, Cat 554413, Lot 65626)
- Anti-Mouse CD11b APC-Cy7 (M1/70, Biolegend)

- Anti-Mouse CD11c PE-Cy7 (N418, Biolegend)
- Anti-Mouse Ly6C PERCP (HK1.4, Biolegend)
- Anti-Mouse MHC-II FITC (M5/11.15.2, Biolegend)
- Anti-Mouse Ly6G APC (1A8, Biolegend)
- Anti-Mouse CD3 FITC (145-2C11, Biolegend)
- Anti-Mouse CD4 APC-Cy7 (GK1.5, BD)
- Anti-Mouse CD8 PERCP (53-6.7, Biolegend)
- Anti-Mouse CD69 PE (H1.2F3, Biolegend)
- Anti-Mouse CD103 BV421 (2E7, Biolegend)
- Anti-Mouse IFN-g APC (XMG1.2, Biolegend)
- Anti-Mouse TNF PE-Cy7 (MP6-XT22, Biolegend)
- Goat Anti-Mouse IgG-HRP (SouthernBiotech, Cat 1030-05, Lot B1411-PD01B)
- Goat Anti-Mouse IgG1-HRP (SouthernBiotech, Cat 1070-05, Lot J6908-T229B)
- Goat Anti-Mouse IgG2c-HRP (SouthernBiotech, Cat 1079-05, Lot D6603-X619)
- Goat Anti-Hamster IgG(H+L)-HRP (SouthernBiotech, Cat 6060-05, Lot I4711-Z990B)
- Goat Anti-Mouse IgG (H + L) Alexa Fluor 594 (Invitrogen, Cat A11032, Lot 1887003)
- Anti-Human CD4 BV605 (RPA-T4, BD),
- Anti-Human CD8 AlexaFluor700 (SK1, Biolegend)
- Anti-Human CD45RO BV786 (UCHL1, BD)
- Anti-Human CD27 APC-Cy7 (O323, Biolegend)
- Anti-Human IFN-γ PE-Cy7 (4S.B3, eBioscience)
- Anti-Human CD3 FITC (UCHT1, BD)
- Anti-Human CD69 BV421 (FN50, Biolegend)
- Anti-Human TNF APC (Mab11, eBioscience)
- Anti-Human HRP FAPON

Validation

Polyclonal antibodies anti-N and anti-RBD used in WB were sera from rabbits immunized with either N or RBD, respectively. All the other antibodies were commercial and validated by the manufacturer. The antibodies for flow cytometry have been tested for mouse splenocytes and were properly titrated before the experiments. The dilutions of antibodies used for WB, ELISA and IFA were recommended by the manufacturer and optimized in our experiments.

-Goat Anti-Mouse IgG-HRP (SouthernBiotech):
Specificity: Reacts with the heavy chains of mouse IgG1, IgG2a, IgG2b, IgG2c, and IgG3
Source: Pooled antisera from goats hyperimmunized with mouse IgG
Cross Adsorption: Mouse IgM and IgA; human immunoglobulins and pooled sera; may react with immunoglobulins from other species
Purification: Affinity chromatography on mouse IgG covalently linked to agarose
Applications: ELISA, FLISA, FC
Working dilutions (ELISA): 1:4,000 - 1:8,000

- Goat Anti-Mouse IgG1-HRP (SouthernBiotech):
Specificity: Reacts with the heavy chain of mouse IgG1
Source: Pooled antisera from goats hyperimmunized with mouse IgG1
Cross Adsorption: Mouse IgG2a, IgG2b, IgG3, IgM, and IgA; human immunoglobulins and pooled sera; may react with immunoglobulins from other species
Purification: Affinity chromatography on mouse IgG1 covalently linked to agarose
Applications: ELISA, FLISA, FC
Working dilutions (ELISA): 1:4,000 - 1:8,000

- Goat Anti-Mouse IgG2c-HRP (SouthernBiotech)
Specificity: Reacts with the heavy chain of C57BL/6 mouse IgG2c
Source: Pooled antisera from goats hyperimmunized with mouse IgG2c
Cross Adsorption: Mouse IgG1, IgG2a, IgG2b, IgG3, IgM, and IgA; may react with immunoglobulins from other species
Purification: Affinity chromatography on mouse IgG2c covalently linked to agarose
Applications: ELISA, FLISA
Working dilutions (ELISA): 1:4,000 - 1:8,000

- Goat Anti-Hamster IgG(H+L)-HRP (SouthernBiotech)
Specificity: Reacts with the heavy and light chains of hamster IgG; may only react with Syrian hamster IgG
Source: Pooled antisera from goats hyperimmunized with hamster IgG
Cross Adsorption: None; may react with immunoglobulins from other species and the light chains of other hamster immunoglobulins
Purification: Affinity chromatography on hamster IgG covalently linked to agarose
Applications: ELISA, FLISA
Working dilutions (ELISA): 1:4,000 - 1:8,000

Eukaryotic cell lines

Policy information about cell lines

Cell line source(s)	Vero E6 cells (ATCC CRL-1586)
Authentication	Cells were low passage cells from ATCC, which authenticates them. Cell morphology and growth was consistent with Vero-E6.
Mycoplasma contamination	Cells were frequently tested for mycoplasma contamination and were negative.
Commonly misidentified lines (See ICLAC register)	None.

Animals and other organisms

Policy information about studies involving animals; ARRIVE guidelines recommended for reporting animal research

Laboratory animals	- Female C57BL/6 mice, 6-10 weeks old<br>- Male and female K18-hACE2 mice, 6-10 weeks old<br>- Female Golden Syrian Hamsters, 6-10 weeks old
Wild animals	The study did not involve wild animals.
Field-collected samples	The study did not include samples collected from the field.
Ethics oversight	The protocols for animal experiments were approved by Fundação Oswaldo Cruz and Universidade Federal de São Paulo Ethics. Commission on Animal Use (CEUA) LW 25/20 and 105/2020, respectively.

Note that full information on the approval of the study protocol must also be provided in the manuscript.

Human research participants

Policy information about studies involving human research participants

Population characteristics	Population characteristics are detailed in table 1. Included subjects were older than 18 years of age and no older than 70 years of both sexes. In Brazil there is a great diversity in genetic origin including African Americans, Asians, South American Indians, Mestizos and Caucasians of European origin, thus our cohort was representative of such diversity. Participants included did not reported any health condition or current treatment considered as a potential bias. We did not anticipate any biases influencing the cohort composition.
Recruitment	All participants were invited to answer a questionnaire and donate blood based on inclusion criteria of each category. Vaccinated participants had complete vaccination with Coronavac within 60 days after booster. Unvaccinated healthy donors (HD) where RT-PCR and antibody test negative, and reported never having had COVID-19. They have not received any vaccine or displayed respiratory symptoms up to 30 days before sampling. Convalescents were unvaccinated before infection and diagnosed by RT-PCR. Potential bias includes gender distribution, since all vaccinated volunteers were health workers that have a higher representation of females. It is also important to notice that all vaccinated volunteers were health workers, whereas HD and convalescent included both health workers and non-health workers. Nevertheless, we believe these potential biases have none or minor effect on results and do not impact on our conclusions.
Ethics oversight	This study was performed under protocols reviewed and approved by the Ethical Committees on Human Experimentation from Fundação Hospitalar do Estado de Minas Gerais (FHEMIG) - CAAE: 43335821.4.0000.5119.

Note that full information on the approval of the study protocol must also be provided in the manuscript.

Flow Cytometry

Plots

Confirm that:

- [x] The axis labels state the marker and fluorochrome used (e.g. CD4-FITC).
- [x] The axis scales are clearly visible. Include numbers along axes only for bottom left plot of group (a 'group' is an analysis of identical markers).
- [x] All plots are contour plots with outliers or pseudocolor plots.
- [x] A numerical value for number of cells or percentage (with statistics) is provided.

Methodology

Sample preparation	Human: PBMCs from healthy donors, convalescents or Coronavac-vaccinated donors were thawed in RPMI 1640 (Sigma-

Aldrich) benzonase nuclease (20 U/mL, Sigma). Cells were plated in complete media (RPMI 1640, 10% FBS, 100 mg/ml streptomycin, 100 U/ml penicillin), rested for 5h and then incubated with positive controls (anti-CD3, 1µg/mL and anti-CD28, 0.5µg/mL, BD), negative controls (SpiN Vehicle) or 20 µg/mL of SpiN in 5% CO_2 at 37°C. After 12h incubation, 2.5µg/mL each of BFA and Monensin was added for additional 6h.

Mice splenocytes: A total of 2 x 10^6 of splenocytes derived from immunized mice were incubated for 18h at 37°C and 5% CO_2 with RPMI 1640 medium alone or containing 10 µg/mL of RBD or N proteins. During the last 6 hours of culture, GolgiStop and GolgiPlug Protein Transport Inhibitors (BD Biosciences) were added to the cell cultures. The splonocytes were then washed with PBS, stained with Live/Dead reagent (Invitrogen) and incubated with FcBlock (BD Biosciences).

Mice lungs: For flow cytometry staining, lungs were excised, minced with scissors, and enzyme-digested using 2 mg/ml of collagenase IV (Sigma) diluted in 1 mL of RPMI. The suspensions were incubated at 37°C for 30 min with regular shake. Tissue fragments were filtered using 50-µm pore size nylon filter Filcon system (BD Biosciences) and then centrifuged. The supernatants were discarded and erythrocytes in the cell pellets were lysed using ACK solution. The remaining cells were resuspended in RPMI 5% FBS, counted in Neubauer chamber, washed with PBS 1x and used for flow cytometry staining.

Instrument

BD LSRFortessa

Software

Collection of data was performed using the Digital DIVA hardware and software and analysis using FlowJo software

Cell population abundance

No cell sorting data.

Gating strategy

Human PBMCs: PBMCs were gated for singlets (FSC-H X FSC-A), lymphocytes (SSC-A x FSC-A), live CD3+ (CD3 x 7AAD), CD3+ CD4+ or CD3+ CD8+ (CD3 x CD4, CD3 x CD8). CD4+ or CD8+ T lymphocytes were characterized in central memory (CD45RO+ CD27+), effector memory (CD45RO+ CD27-), effector (CD45RO- CD27-) and naive (CD45RO- CD27+).

Mice splenocytes: Splenocytes were gated for singlets (FSC-H X FSC-A), lymphocytes (SSC-A x FSC-A), live CD3+ (CD3 x Live/Dead), CD4+ or CD8+ (SSC-A x CD4, SSC-A x CD8), IFN-γ+ (SSC-A x IFN-γ) and with CD44 x CD62L they were separated into naïve (CD44- CD62L+), effector/effector memory (CD44+ CD62L-) and central memory (CD44+ CD62L+).

Mice lungs: Lungs cells were gated for singlets (FSC-H X FSC-A), live (SSC-A x Live/Dead), lymphocytes (SSC-A x FSC-A), CD3+ CD4+ or CD3+ CD8+ (CD3 x CD4, CD3 x CD8). CD4+ or CD8+ T lymphocytes were characterized in CD69+ CD103+ tissue-resident memory (Trm) cells by Boolean analysis “AND gate” (CD69+ and CD103+), Trm IFN-γ+ (CD69+ CD103+ and IFN-γ+), Trm TNF+ (CD69+ CD103+ and TNF+).

☒ Tick this box to confirm that a figure exemplifying the gating strategy is provided in the Supplementary Information.
